# Endoscopic discectomy for L4-L5 disc herniation: A meta-analysis comparing transforaminal and interlaminar approaches

**DOI:** 10.1016/j.bas.2026.105925

**Published:** 2026-01-05

**Authors:** Ralph Maroun, Youssef Jamaleddine, Chahine Assi, Ramzi Moucharafieh, Mohammad Badra

**Affiliations:** a Department of Orthopedic Surgery, Lebanese University, Beirut, Lebanon; b Department of Orthopedic Surgery, Lebanese American University Medical Center‐Rizk Hospital, Lebanon; c Department of Orthopedic Surgery, Gilbert and Rose-Marie Chagoury School of Medicine, Lebanese American University, Lebanon; d Department of Orthopedics and Traumatology, Clemenceau Medical Center, Lebanon; e Department of Orthopedic Surgery, Faculty of Medicine, Balamand University, Lebanon

**Keywords:** Lumbar disc herniation, Endoscopic discectomy, Transforaminal, Interlaminar, L4-L5 disc

## Abstract

**Background:**

Lumbar disc herniation can be debilitating. Percutaneous endoscopic discectomy (PED) is an emerging minimally invasive alternative to microdiscectomy, performed through either an interlaminar (IL) or transforaminal (TF) approach.

**Research question:**

There is existing evidence comparing these two approaches for managing the most common herniation level, L4-L5, but it lacks consistency and clear conclusions. Therefore, a meta-analysis is necessary to determine if one approach is superior to the other.

**Methods:**

Medline, Cochrane, and Google Scholar (pages 1–20) were searched until August 1, 2025, following PRISMA guidelines. The data extracted included overall complications, reoperation rates, operative time, length of stay (LOS), and improvements in patient-reported outcome measures (PROMs) at least one year after surgery.

**Results:**

Six studies involving 456 patients (276 TF; 180 IL) were included. No significant differences were found between the two approaches regarding overall complications (OR = 1.81; 95 % CI: 0.51–6.44; p = 0.36), reoperation rates (OR = 2.10; 95 % CI: 0.38–11.70; p = 0.40), operative time (MD = 0.73; 95 % CI: −14.83–16.29; p = 0.93), or LOS (MD = 0.03; 95 % CI: −0.13–0.19; p = 0.69). Similarly, improvements in ODI (MD = −1.06; 95 % CI: −2.63–0.52; p = 0.19), back pain (MD = 0.29; 95 % CI: −0.61–1.19; p = 0.53), and leg pain (MD = −0.44; 95 % CI: −1.19–0.31; p = 0.25) showed no significant differences.

**Discussion and conclusion:**

Both approaches produce similar results regarding overall complications, reoperation rates, operative time, LOS, and PROMs. The choice of approach should thus be based on surgeon experience, patient-specific anatomy, and resource availability.

## Highlights

• Interlaminar and transforaminal approaches show similar complication rates.
• Operative time is similar between the two surgical approaches.
• Hospital length of stay and reoperation rates do not differ between approaches.
• Patient-reported outcomes are similar for both approaches.
• Choose approach based on surgeon experience, anatomy, and resources.

## 1 Introduction

Lumbar disc herniation (LDH) is a primary cause of low-back pain and sciatica, causing significant social, functional, emotional, and economic burdens on patients (Zhang et al., 2023a; Taşkaya et al., 2024; Kögl et al., 2024; Zhou et al., 2024). Conservative management is the initial standard of care for most cases; surgery is reserved for red-flag presentations or persistent, disabling symptoms despite adequate nonoperative treatment (Zhang et al., 2023a; Al Qaraghli and De Jesus, 2025). For non-emergent LDH cases, the decision on treatment modality is personalized by shared decision making, supported by clinical evaluation, symptom duration, and the patient's preferences (Zhang et al., 2023a; Al Qaraghli and De Jesus, 2025).

In the United States, lumbar discectomy for the treatment of LDH is very common (Blamoutier, 2013; Weinstein et al., 2006). Currently, microdiscectomy, known for its smaller incision and tissue-sparing potential compared to open discectomy, is widely regarded as a leading approach in lumbar discectomy (Dowling et al., 2025). However, the percutaneous endoscopic discectomy (PED) has been rapidly gaining popularity with excellent outcomes (Jamaleddine et al., 2025). PED offers a faster post-operative recovery and a high tissue-sparing potential; it also reduces blood loss and operative time while maintaining similar and sometimes superior outcomes to microdiscectomy (Aiyer et al., 2022; Muthu et al., 2021; Gadjradj et al., 2021; Gibson et al., 2017). Moreover, PED can be performed using two major approaches: the interlaminar approach (IL) and the transforaminal approach (TF), each with its own benefits and drawbacks (Pruttikul et al., 2025).

More than 90 % of LDHs occur at L4-L5 or L5-S1, and for considerable anatomical considerations, L4-L5 LDHs are primarily treated with the TF approach, whereas L5-S1 LDHs are done through an IL PED (Al Qaraghli and De Jesus, 2025; Xu et al., 2024; Zhao et al., 2022). Nonetheless, most systematic studies comparing IL PED and TF PED are limited to the L5-S1 level (Pruttikul et al., 2025; Zhao et al., 2022). As for L4-L5, conflicting results exist between the studies. For instance, one study found that the IL approach had better pain and ODI post-operatively than the TF approach (Zhao et al., 2022). Moreover, some studies reported a shorter operative time for the IL approach (Xu et al., 2024; Zhao et al., 2022; Huang et al., 2021). Bamrungthin et al. reported a lower rate of complications in the IL approach. Conversely, some studies showed no significant difference in postoperative PROMs between the two approaches (Pruttikul et al., 2025; Bamrungthin, 2020). Also, some studies reported a longer operative time for the IL approach (Pruttikul et al., 2025; Takebayashi et al., 2023). While some other studies reported no difference in complication rate between the two groups (Xu et al., 2024; Zhao et al., 2022; Takebayashi et al., 2023). These conflicting results highlight the importance of consolidating the data in a meta-analysis to clarify subtle differences, guide better surgical decision-making, and potentially determine if patients with L4-L5 LDH would benefit more from one approach over the other.

## 2 Material and methods

### 2.1 Search strategy

**Fig. 1:**
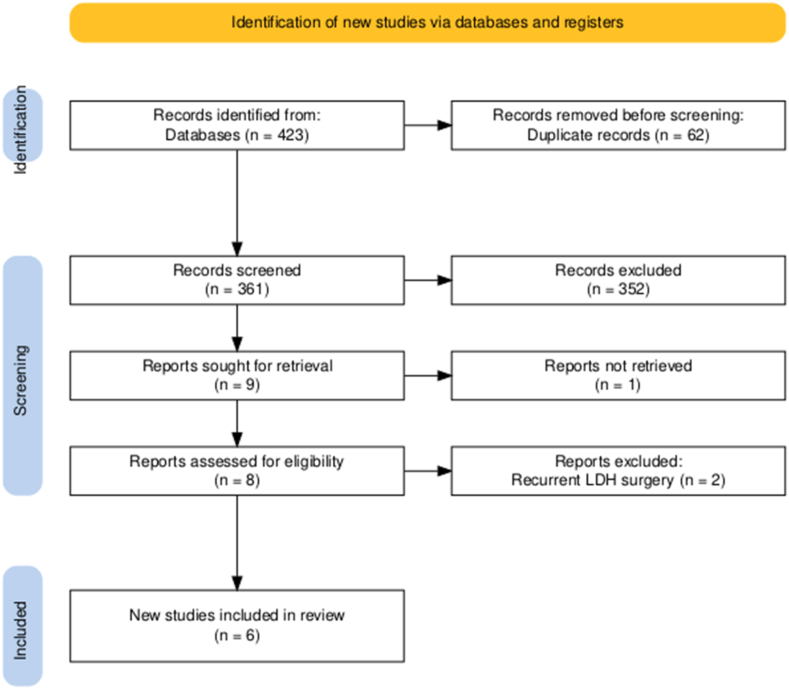
PRISMA flow diagram of the included studies.

This meta-analysis was conducted following the Preferred Reporting Items for Systematic reviews and Meta-Analyses (PRISMA) guidelines (Page et al., 2021). The Cochrane Library, Medline, and Google Scholar (pages 1–20) were accessed and explored from inception through August 1, 2025. The following keywords were used and combined using Boolean operators “AND” and “OR” to identify articles comparing transforaminal endoscopic lumbar discectomy to interlaminar endoscopic lumbar discectomy for L4-L5 LDH: “endoscopic”, “transforaminal”, “interlaminar”, “disk∗”, and “disc∗”. Reference lists from included studies were also searched for additional articles. The process is summarized in the PRISMA flowchart (Fig. 1).

### 2.2 Eligibility criteria

Inclusion criteria consisted of English and non-English studies comparing interlaminar and transforaminal endoscopic lumbar discectomy for the L4-L5 LDH. Non-comparative studies, case reports, review articles, and editorial commentaries were excluded. Additionally, database studies were also excluded to avoid overlap of patients. Studies that did not include at least one outcome of interest were also excluded.

Eligibility of the included studies was determined by two reviewers independently. Disagreements when present were discussed and resolved by a third senior independent author.

### 2.3 Data extraction

Microsoft Excel spreadsheet (Version, 2021; Microsoft) was used for the extraction and tabulation of study characteristics, surgery-related outcomes, PROMs, overall complications, and reoperations.

Study characteristics included sample sizes by the approach of endoscopy, study design, study's country of execution, patient demographics such as mean age, sex, and mean of last patient follow-up time. Surgery-related outcomes included operative time (reported in minutes) and length of stay (LOS) (reported in days). PROMs included Oswestry Disability Index (ODI) and Visual Analogue Scale (VAS) for back and leg pain, for which the mean improvement from preoperative to postoperative (at ≥ 1 year follow-up) levels was calculated and used for analysis. Overall complications consisted of minor and major adverse events, excluding the number of reoperations or revision surgeries. Reoperations were defined as any revision surgery performed at the index level following the initial procedure.

### 2.4 Risk of bias assessment

Two authors independently assessed, using The Risk Of Bias In Nonrandomized Studies-of Interventions (ROBINS-I) tool, the risk of bias for included studies with a retrospective design (Sterne et al., 2016). A third independent author resolved the disagreement. Additionally, the revised tool RoB-2 was used to evaluate the risk of bias in the included prospective, randomized study (Pruttikul et al., 2025; Sterne et al., 2019).

### 2.5 Statistical analysis

Review Manager 5.4 (The Cochrane Collaboration, 2020) was implemented for the statistical analysis. Mean differences (MD) with 95 % CI were used for continuous data, while the odds ratio (OR) was utilized for dichotomous data. Heterogeneity was evaluated by Q tests and *I*^*2*^ statistics. If considerable heterogeneity was indicated by *p* ≤ 0.05 or *I*^*2*^> 50 %, a random-effects model was used. Otherwise, the fixed-effect model was implemented. A statistically significant result is shown by *p* ≤ 0.05. For Zhao et al., in which the Visual Analog Scale (VAS) was not specified as assessing either leg or back pain, the VAS data were included in both the leg pain and back pain forest plots. Sensitivity analyses were conducted to determine the impact of this inclusion (Zhao et al., 2022).

## 3 Results

### 3.1 Characteristics of the included studies

**Fig. 2:**
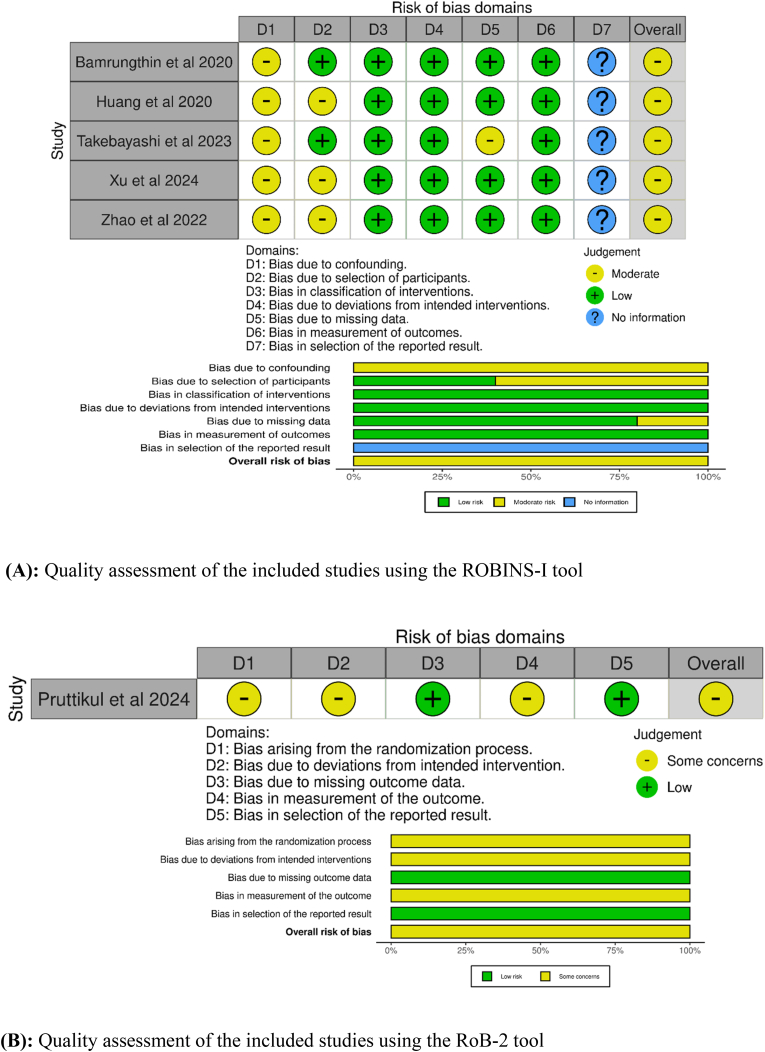
Quality assessment of the included studies.

Six studies met the inclusion criteria (Pruttikul et al., 2025; Xu et al., 2024; Zhao et al., 2022; Huang et al., 2021; Bamrungthin, 2020; Takebayashi et al., 2023). These studies included 456 patients, with 276 in the TF group with a mean age of 42.1, and 180 patients in the IL group with a mean age of 44.4. The main characteristics of the included studies are summarized in Table 1. The results of the risk-of-bias assessment of the included studies are summarized in Fig. 2A and B.

**Table 1:** Main characteristics of included studies (TF = transforaminal; IL = Interlaminar; LDH = lumbar disc herniation; N/A = Not Available).

Paper	Country	Number of patients		Mean age (years)		Type of study	Latest Follow-up period (months)		Overall complications		Reoperation rate	
		TF	IL	TF	IL		TF	IL	TF	IL	TF	IL
Bamrungthin et al. 2020^18^	Thailand	33	52	45.1	43.7	Retrospective	24		−6 Pain −9 Dysesthesia −3 Transient motor deficit	−4 pain −3 dysesthesia −1 Transient motor deficit	6	2
Huang et al. 2020^17^	China	37	40	43.7	45.7	Retrospective	20.5	22.6	−3 recurrent LDH	−2 transient skin paresthesia −2 recurrent LDH	N/A	
Pruttikul et al. 2024^14^	Thailand	30	30	37.7	39.3	Prospective, Randomized	24		−4 recurrent LDH −9 dysesthesia −2 motor weakness	−2 recurrent LDH −1 dysesthesia −1 dural tear	4	1
Takebayashi et al., 2023^19^	Japan	105	19	44.8	46.4	Retrospective	24		−5 recurrent LDH	−2 recurrent LDH	5	2
Xu et al., 2024^15^	China	17	17	33.3	38.1	Retrospective	12		0	−1 transient numbness	N/A	
Zhao et al., 2022^16^	China	54	22	47.8	53.3	Retrospective	20.8	19.3	0	0	N/A

### 3.2 Complications and reoperation

**Fig. 3:**
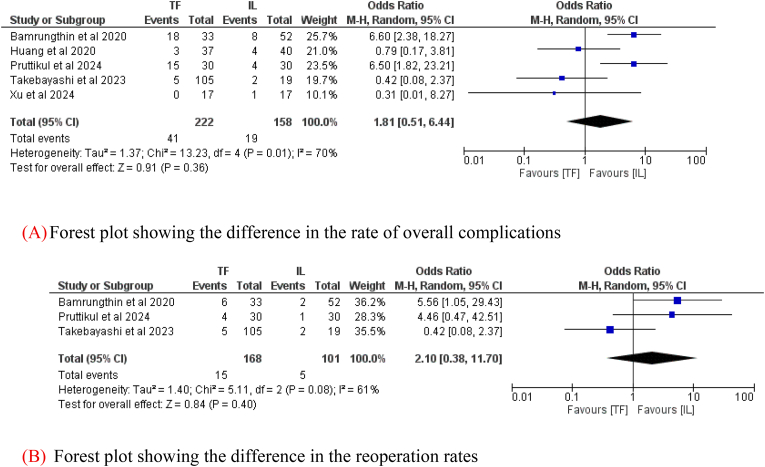
(A): Forest plot showing the differencein the rate of overall complication. (B): Forest plot showing the difference in the reoperation rates.

Five studies, including 380 patients, reported data about overall complications (222 in the TF group and 158 in the IL group). There was no difference in the rate of reported overall complications (Odds Ratio = 1.81; 95 % CI: 0.51–6.44, *p* = 0.36, Fig. 3A) between the two groups. In addition, three studies, including 269 patients (168 in the TF group and 101 in the IL group), reported data on reoperations. Similarly, the pooled analysis demonstrated no significant difference in reoperation rates between TF and IL approaches (Odds Ratio = 2.10; 95 % CI: 0.38–11.70; p = 0.40, Fig. 3B).

### 3.3 Surgery-related outcomes

**Fig. 4:**
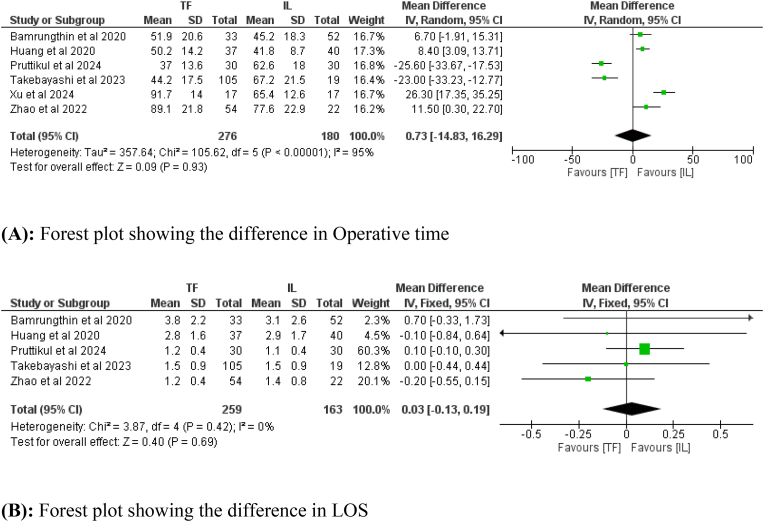
(A): Forest plot showing the difference in Operative time. (B): Forest plot showing the difference in LOS.

Six studies, including 456 patients, reported data about operative time (276 in the TF group and 180 in the IL group), and five studies including 422 patients, reported data about LOS (259 in the TF group and 163 in the IL group). There was no difference in operative time (Mean difference = 0.73; 95 % CI: −14.83– 16.29, *p* = 0.93, Fig. 4A), and LOS (Mean difference = 0.03; 95 % CI: −0.13– 0.19, *p* = 0.69, Fig. 4B) between the two groups.

### 3.4 PROMs

**Fig. 5:**
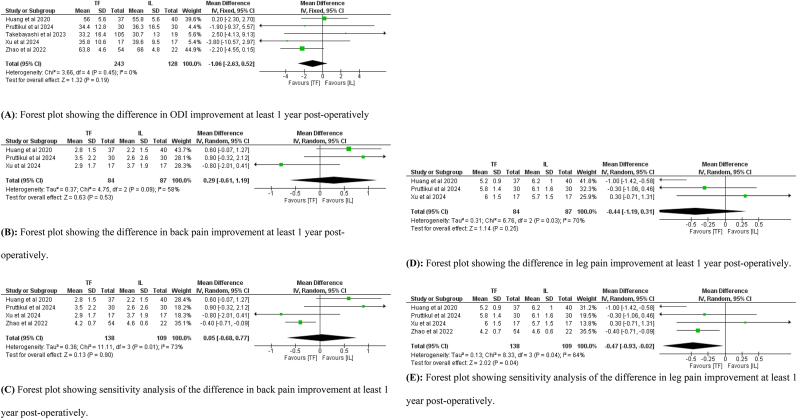
(A): Forest plot showing the difference in ODI improvement at least 1 year post-operatively. (B): Forest plot showing the difference in back pain improvement at least 1 year post-operatively. (C) Forest plot showing sensitivity analysis of the difference in back pain improvement at least 1 year post-operatively. (D): Forest plot showing the difference in leg pain improvement at least 1 year post-operatively. (E): Forest plot showing sensitivity analysis of the difference in leg pain improvement at least 1 year post-operatively.

Five studies, including 371 patients, reported data on the improvement in ODI (243 in the TF group and 128 in the IL group), three studies including 171 patients, reported data about the improvement in back and leg pain (84 in the TF group and 87 in the IL group). There was no significant difference between the two groups in ODI improvement (Mean difference = -1.06; 95 % CI: −2.63– 0.52, *p* = 0.19, Fig. 5A). No significant difference was found between the two groups in back pain improvement (Mean difference = 0.29; 95 % CI: −0.61– 1.19, *p* = 0.53, Fig. 5B). Additionally, inclusion of the study that reported unspecified area pain did not significantly alter the result (Mean difference = 0.05; 95 % CI: −0.68– 0.77, *p* = 0.9, Fig. 5C). As for improvement in leg pain, there was no significant difference between the two groups (Mean difference = -0.44, 95 % CI: 1.19 – 0.31, *p* = 0.25, Fig. 5D). However, when the study reporting unspecified area pain was included, TF significantly reported better leg pain improvement (Mean difference = -0.47, 95 % CI: 0.93 to −0.02, *p* = 0.04, Fig. 5E).

## 4 Discussion

Percutaneous endoscopic discectomy is increasingly recognized as one of the most common techniques for managing LDHs (Zhao et al., 2022; Mahesha, 2017). This procedure is widely performed through either an IL approach or a TF approach for the two most common herniations at L4-L5 and L5-S1 (Xu et al., 2024; Zhao et al., 2022). Depending on the surgeon's preference and anatomical variations, the transforaminal approach is mainly used for treating L4-L5 disc herniation, while the interlaminar approach is employed for L5-S1 (Xu et al., 2024; Zhao et al., 2022). However, meta-analyses comparing the outcomes of the two approaches have been limited to herniation at the L5-S1 level; thus, motivating our study (Zhu et al., 2024; Chen et al., 2018). The results of our study show that treating an L4-L5 disc herniation with either approach results in similar outcomes in terms of overall complications, reoperation rates, operative time, LOS, and improvement in back and leg pain at 1 year.

Patients undergoing PED for L4-L5 disc herniation through either an IL or transforaminal approach showed no difference in the overall complication rate. Similar results were also observed by Jitpakdee et al., a meta-analysis that compared outcomes between IL and TF PED for LDHs across different lumbar levels, attributing the similarity to the surgeons' learning curve (Jitpakdee et al., 2023). Additionally, Ju et al. found no difference in the complication rates between the two approaches in endoscopic surgery, regardless of spinal level (Ju and Lee, 2023). This may also be explained by the PED procedure's low complication rates reported in the literature, which makes small sample studies underpowered to detect actual differences. (Yang et al., 2022; Li et al., 2022; Chen et al., 2020). Similarly, our meta-analysis found no significant difference in reoperation rates between the TF and IL approaches, consistent with previous studies comparing transforaminal and interlaminar lumbar endoscopic discectomy for disc herniations across different lumbar levels (Jamaleddine et al., 2025).

Regarding LOS and operative time, our study found no difference between the two approaches. The analysis of operative time shows conflicting results across studies; Bamrungthin et al. show no difference between the two groups, while Huang et al. report a shorter operative time in the IL approach group (Huang et al., 2021; Bamrungthin, 2020). Conversely, Pruttikul et al. and Takebayashi et al. observed a shorter operative time in the TF approach group (Pruttikul et al., 2025; Takebayashi et al., 2023). This may not be surprising, as our pooled analysis of operative time indicates a high level of heterogeneity, I^2^ = 95 %. The heterogeneity among included studies can arise from differences in surgeons, their learning curves, and their indications by approach. On the other hand, Jitpakdee et al. observed similar findings in the LOS analysis, aligning with the results of studies included in our review (Pruttikul et al., 2025; Xu et al., 2024; Zhao et al., 2022; Huang et al., 2021; Bamrungthin, 2020; Takebayashi et al., 2023; Jitpakdee et al., 2023). This can be attributed to the minimally invasive nature of the procedure, regardless of the approach used, as it involves smaller incisions, less muscle trauma, and quicker recovery after surgery (Aiyer et al., 2022; Muthu et al., 2021; Gadjradj et al., 2021; Gibson et al., 2017).

Our analysis also revealed no significant difference in ODI improvement or leg and back pain at ≥ 1 year when comparing the two approaches. These findings align with earlier research showing similar effectiveness comparing IL and TF PED, for L4-L5 LDH alone, L5-S1 LDH alone, or different level lumbar herniations (Pruttikul et al., 2025; Xu et al., 2024; Bamrungthin, 2020; Takebayashi et al., 2023; Chen et al., 2018; Li et al., 2024; Zhang et al., 2023b). However, Zhao et al. measured pain using a visual analogue scale, without specifying whether it was for back or leg pain (Zhao et al., 2022). Therefore, this data was excluded from the primary pooled analysis of back and leg pain to avoid bias. A sensitivity analysis was conducted, adding this study to both pooled analyses of leg and back pain, showing a particularly significant difference in leg pain improvement, favoring the transforaminal approach. This sensitivity analysis emphasizes the importance of clearly defining outcomes, as it can influence the pooled results. Finally, the lack of a clear advantage regarding disability and pain improvement in our review suggests that the choice of surgical approach should primarily depend on the surgeon's expertise, accessible equipment, patient-specific anatomical factors, or financial considerations, rather than anticipated variations in clinical outcomes.

### 4.1 Strengths and limitations

Several potential limitations exist in this meta-analysis. The possible variation in surgical techniques, post-operative care, and the surgeons’ learning curve in this emerging minimally invasive field may have introduced heterogeneity into our analysis. However, when the pooled analysis showed high heterogeneity, a random-effects model was used, following the Cochrane handbook for systematic reviews and meta-analysis. The retrospective design of most of the included studies introduces bias inherent to this type of research; additional high-quality prospective comparative studies are needed to reduce bias and strengthen the evidence base in this area. Additionally, the limited number of studies available for inclusion prevented sub-analyses based on different types of lumbar herniation. Despite these limitations, this meta-analysis is the first to compare outcomes between transforaminal and interlaminar endoscopic approaches for L4-L5 lumbar herniation discectomy.

## 5 Conclusion

Overall, for patients with an L4-L5 disc herniation, interlaminar and transforaminal approaches in endoscopic discectomy present similar operative time, LOS, risk of complications, reoperation rates, and improvement in pain and disability. These results emphasize that the choice of approach should mainly be guided by factors specific to surgeons and patients rather than predicted advantages in clinical outcomes.

## Conflict of interest

The authors declare that they have no known competing financial interests or personal relationships that could have appeared to influence the work reported in this paper.
